# Editorial: Action effects in perception and action

**DOI:** 10.3389/fpsyg.2013.00223

**Published:** 2013-05-03

**Authors:** Roland Pfister, Markus Janczyk, Wilfried Kunde

**Affiliations:** Department of Psychology III, Julius Maximilians University of WürzburgWürzburg, Germany

Good scientific theories should be simple, valid, and stimulating. It seems that ideomotor theory, which has been the core theme behind the research topic on “*Action Effects in Perception and Action,”* has done a fairly good job in terms of these three criteria. First, it is rather simple: goal-directed actions are assumed to be selected and addressed by anticipating their sensory consequences; crucially, learned bidirectional associations between sensory representations and motor commands ensure that these anticipations eventually result in overt behavior. Secondly, numerous observations comply with its basic predictions, derived from philosophical analyses of the nineteenth century (cf. Stock and Stock, 2004; Pfister and Janczyk, 2012). Accordingly, the validity of ideomotor theory has been documented by extensive empirical research over the last decades (e.g., Elsner and Hommel, 2001; Hommel et al., 2001; Kunde, 2001; see also Shin et al., 2010).

Thirdly, ideomotor theory (still) seems to stimulate contemporary research. Otherwise the impressive range of topics that have come together in the present research topic can hardly be explained. These topics range from investigations of how attention and perception are modulated by intentions and expectations (Kemper et al., 2012; Wykowska and Schubö, 2012), to applied settings such as aging and tool-use (Sutter et al., 2012), task-switching (Lukas et al., 2013), to social influences on action coding (Colzato et al., 2012; Nishimura and Michimata, 2013) and a developmental perspective on action effects in object manipulation (Knudsen et al., 2012). These new perspectives are backed up by studies on two prevailing questions in ideomotor research: The formation of action-effect associations (Herwig and Waszak, 2012; Janczyk et al., 2012; Ruge et al., 2012)—including a first step toward addressing individual differences in ideomotor learning (Muhle-Karbe and Krebs, 2012)—and the role of such associations for action control (Gaschler and Nattkemper, 2012; Walter and Rieger, 2012; Ziessler et al., 2012).

Furthermore, three notable articles explore theoretical refinements of ideomotor theory by addressing the virtue of visuomotor priming for ideomotor research (Thomaschke, 2012), hierarchical coding of action-effect relations (Ondobaka and Bekkering, 2012) and computational constraints for ideomotor theory (Herbort and Butz, 2012).

In the light of these and other recent empirical and theoretical advances (cf. Shin et al., 2010), it seems as if twenty-first century ideomotor theory accounted for almost all areas of cognitive psychology. On careful consideration, however, it also seems as if a particular area is still underrepresented in the ideomotor community, and this area is the concept of working memory. Whereas there are a several short hints to “memory traces” or “long-term memory” throughout the articles of the research topic, the concept of working memory is mentioned only a single time (Thomaschke, 2012, p. 4). Arguably, however, anticipated action effect must be represented somewhere in the cognitive architecture—and working memory appears a likely place for these representations. In our view, this state of affairs is indicative of the current theoretical state and calls for a better exchange between the respective scientific communities.

Similarly, while the sketched developments and directions are admirable on their own, they also pose a new challenge for scholars of action and perception. This challenge relates to an explicit treatment of the relations—commonalities and differences—of the ideomotor approach to other general frameworks for action and perception. For instance, the neuroscientific approaches of predictive coding (Rao and Ballard, 1998), the Bayesian brain (Knill and Pouget, 2004), and the free-energy principle (Friston, 2010) seem to share many features with the principles of effect-based action control even though the different accounts are rarely discussed in the same place (and are nourished by distinct scientific communities). In the same vein, relations to accounts for the perception of self-generated action effects (Haggard et al., 2002; Baess et al., 2011; Moore and Obhi, 2012) need a more explicit treatment, and so do the relations to mathematical models of human motor control (Wolpert and Ghahramani, 2000).

In the meantime, we would like to thank all authors who joined the enterprise of this research topic, and all reviewers who commented on the presented papers. It was a pleasant enterprise from beginning to end, i.e., from sending out the first invitations up to the final, joint action effect which is the research topic itself.

## References

[B1] Baess P. Horváth J. Jacobsen T. Schröger E. (2011). Selective suppression of self-initiated sounds in an auditory stream: an ERP study. Psychophysiology 48, 1276–1283 10.1111/j.1469-8986.2011.01196.x 21449953

[B2] ^*^Colzato L. S. de Bruijn E. R. A. Hommel B. (2012). Up to “me” or up to “us”? The impact of self-construal priming on cognitive self-other integration. Front. Psychol. 3:341 10.3389/fpsyg.2012.00341 23049518 PMC3442283

[B3] Elsner B. Hommel B. (2001). Effect anticipation and action control. J. Exp. Psychol. Hum. Percept. Perform. 27, 229–240 10.1037/0096-1523.27.1.229 11248937

[B4] Friston K. (2010). The free-energy principle: a unified brain theory? Nat. Rev. Neurosci. 11, 127–138 10.1038/nrn2787 20068583

[B5] ^*^Gaschler R. Nattkemper D. (2012). Instructed task demands and utilization of action effect anticipation. Front. Psychol. 3:578 10.3389/fpsyg.2012.00578 23431066 PMC3577050

[B6] Haggard P. Clark S. Kalogeras J. (2002). Voluntary action and conscious awareness. Nat. Neurosci. 5, 382–385 10.1038/nn827 11896397

[B7] ^*^Herbort O. Butz M. V. (2012). Too good to be true? Ideomotor theory from a computational perspective. Front. Psychol. 3:494 10.3389/fpsyg.2012.00494 23162524 PMC3495337

[B8] ^*^Herwig A. Waszak F. (2012). Action-effect bindings and ideomotor learning in intention- and stimulus-based actions. Front. Psychol. 3:444 10.3389/fpsyg.2012.00444 23112785 PMC3481004

[B9] Hommel B. Müsseler J. Aschersleben G. Prinz W. (2001). The theory of event coding: a framework for perception and action. Behav. Brain Sci. 24, 849–878 12239891 10.1017/s0140525x01000103

[B10] ^*^Janczyk M. Heinemann A. Pfister R. (2012). Instant attraction: immediate action-effect bindings occur for both, stimulus- and goal-driven actions. Front. Psychol. 3:446 10.3389/fpsyg.2012.00446 23112787 PMC3481005

[B11] ^*^Kemper M. Umbach V. J. Schwager S. Gaschler R. Frensch P. A. Stürmer B. (2012). What I say is what I get: stronger effects of self-generated vs. cue-induced expectations in event-related potentials. Front. Psychol. 3:562 10.3389/fpsyg.2012.00562 23403896 PMC3565970

[B12] Knill D. C. Pouget A. (2004). The Bayesian brain: the role of uncertainty in neural coding and computation. Trends Neurosci. 27, 712–719 10.1016/j.tins.2004.10.007 15541511

[B13] ^*^Knudsen B. Henning A. Wunsch K. Weigelt M. Aschersleben G. (2012). The end-state comfort effect in 3- to 8-year-old children in two object manipulation tasks. Front. Psychol. 3:445 10.3389/fpsyg.2012.00445 23112786 PMC3482869

[B14] Kunde W. (2001). Response-effect compatibility in manual choice reaction tasks. J. Exp. Psychol. Hum. Percept. Perform. 27, 387–394 10.1037/0096-1523.27.2.387 11318054

[B15] ^*^Lukas S. Philipp A. M. Koch I. (2013). The influence of action effects in task-switching. Front. Psychol. 3:595 10.3389/fpsyg.2012.00595 23441055 PMC3578419

[B16] Moore J. W. Obhi S. S. (2012). Intentional binding and the sense of agency: a review. Conscious. Cogn. 21, 546–561 10.1016/j.concog.2011.12.002 22240158

[B17] ^*^Muhle-Karbe P. S. Krebs R. M. (2012). On the influence of reward on action-effect binding. Front. Psychol. 3:450 10.3389/fpsyg.2012.00450 23130005 PMC3487417

[B18] ^*^Nishimura A. Michimata C. (2013). Pointing hand stimuli induce spatial compatibility effects and effector priming. Front. Psychol. 4:219 10.3389/fpsyg.2013.00219 PMC3636509 23637688

[B19] ^*^Ondobaka S. Bekkering H. (2012). Hierarchy of idea-guided action and perception-guided movement. Front. Psychol. 3:579 10.3389/fpsyg.2012.00579 23423700 PMC3573717

[B20] Pfister R. Janczyk M. (2012). Harleß' apparatus of will: 150 years later. Psychol. Res. 76, 561–565 10.1007/s00426-011-0362-3 21748464 PMC3419348

[B21] Rao R. P. Ballard D. H. (1998). Predictive coding in the visual cortex: a functional interpretation of some extra-classical receptive field effects. Nat. Neurosci. 2, 79–87 10.1038/4580 10195184

[B22] ^*^Ruge H. Krebs R. M. Wolfensteller U. (2012). Early markers of ongoing action-effect learning. Front. Psychol. 3:522 10.3389/fpsyg.2012.00522 23205016 PMC3506999

[B23] Shin Y. K. Proctor R. W. Capaldi E. J. (2010). A review of contemporary ideomotor theory. Psychol. Bull. 136, 943–974 10.1037/a0020541 20822210

[B24] Stock A. Stock C. (2004). A short history of ideo-motor action. Psychol. Res. 68, 176–188 10.1007/s00426-003-0154-5 14685855

[B25] ^*^Sutter C. Ladwig S. Oehl M. Müsseler J. (2012). Age effects on controlling tools with sensorimotor transformations. Front. Psychol. 3:573 10.3389/fpsyg.2012.00573 23293617 PMC3536330

[B26] ^*^Thomaschke R. (2012). Investigating ideomotor cognition with motorvisual priming paradigms: key findings, methodological challenges, and future directions. Front. Psychol. 3:519 10.3389/fpsyg.2012.00519 23189067 PMC3505020

[B27] ^*^Walter A. M. Rieger M. (2012). Similar mechanisms of movement control in target- and effect-directed actions toward spatial goals? Front. Psychol. 3:539 10.3389/fpsyg.2012.00539 23230426 PMC3515765

[B28] Wolpert D. M. Ghahramani Z. (2000). Computational principles of movement neuroscience. Nat. Neurosci. 3, 1212–1217 10.1038/81497 11127840

[B29] ^*^Wykowska A. Schubö A. (2012). Action intentions modulate allocation of visual attention: electrophysiological evidence. Front. Psychol. 3:379 10.3389/fpsyg.2012.00379 23060841 PMC3464057

[B30] ^*^Ziessler M. Nattkemper D. Vogt S. (2012). The activation of effect codes in response preparation: new evidence from an in direct priming paradigm. Front. Psychol. 3:585 10.3389/fpsyg.2012.00585 23293623 PMC3533231

